# Incorporating sampling weights into robust estimation of Cox proportional hazards regression model, with illustration in the Multi-Ethnic Study of Atherosclerosis

**DOI:** 10.1186/s12874-020-00945-9

**Published:** 2020-03-14

**Authors:** Colleen M. Sitlani, Thomas Lumley, Barbara McKnight, Kenneth M. Rice, Nels C. Olson, Margaret F. Doyle, Sally A. Huber, Russell P. Tracy, Bruce M. Psaty, Joseph A. C. Delaney

**Affiliations:** 1 Department of Medicine, Cardiovascular Health Research Unit, University of Washington, 1730 Minor Ave, Suite 1360, Seattle, 98101 WA USA; 2 Department of Statistics, University of Auckland, Auckland, New Zealand; 3 Department of Biostatistics, University of Washington, Seattle, WA USA; 4 Department of Pathology and Laboratory Medicine, Robert Larner, M.D. College of Medicine, University of Vermont, Burlington, VT USA; 5 Department of Biochemistry, University of Vermont, Burlington, VT USA; 6 Department of Epidemiolgy, University of Washington, Seattle, WA USA; 7 Department of Health Services, University of Washington, Seattle, WA USA; 8 Kaiser Permanente Washington Health Research Institute, Seattle, WA USA; 9 College of Pharmacy, University of Manitoba, Winnipeg, MB Canada

**Keywords:** Cox regression, Sampling weights, Case-cohort design, Robust regression, Immune cell traits

## Abstract

**Background:**

Cox proportional hazards regression models are used to evaluate associations between exposures of interest and time-to-event outcomes in observational data. When exposures are measured on only a sample of participants, as they are in a case-cohort design, the sampling weights must be incorporated into the regression model to obtain unbiased estimating equations.

**Methods:**

Robust Cox methods have been developed to better estimate associations when there are influential outliers in the exposure of interest, but these robust methods do not incorporate sampling weights. In this paper, we extend these robust methods, which already incorporate influence weights, so that they also accommodate sampling weights.

**Results:**

Simulations illustrate that in the presence of influential outliers, the association estimate from the weighted robust method is closer to the true value than the estimate from traditional weighted Cox regression. As expected, in the absence of outliers, the use of robust methods yields a small loss of efficiency. Using data from a case-cohort study that is nested within the Multi-Ethnic Study of Atherosclerosis (MESA) longitudinal cohort study, we illustrate differences between traditional and robust weighted Cox association estimates for the relationships between immune cell traits and risk of stroke.

**Conclusions:**

Robust weighted Cox regression methods are a new tool to analyze time-to-event data with sampling, e.g. case-cohort data, when exposures of interest contain outliers.

## Background

Cox proportional hazards regression models [1] are widely used for analysis of time-to-event data. Modifications of traditional Cox models have been developed to accommodate several important scenarios, including data sampled from a bigger population of interest and data containing influential outliers. In the context of data sampling, estimates can be weighted by the inverse sampling probability [2]. To reduce the impact of violation of model assumptions, several robust methods have been proposed [3–6]. One robust method focuses on robustness to variation in proportional hazards over time [5, 7], and incorporates sampling weights. However, a related robust method that focuses on robustness to influential outliers [3, 8, 9] does not incorporate sampling weights.

One context in which sampling weights play an important role is the case-cohort design, a strategy used to maximize power for a primary outcome of interest and, at the same time, facilitate the analysis of multiple secondary outcomes. When measurement of an exposure of interest in all members of a cohort is not feasible, measuring it in a random ‘cohort’ of participants, plus all additional ‘cases’ who experience the primary outcome can be an efficient study design [10]. Several methods exist for analyzing case-cohort data, but one relatively simple one involves use of inverse sampling weights [11]. Accounting for the sampling scheme is crucial in obtaining unbiased estimates that reflect population-level associations between exposure and outcome.

One example of an ongoing case-cohort study in which outliers play an important role is a study of immune cell traits analyzed in a sub-cohort of the Multi-Ethnic Study of Atherosclerosis (MESA) longitudinal cohort study [12] that includes all cases of angina and myocardial infarction (MI). A number of lymphocyte and monocyte subsets were measured in this sub-cohort, using methods similar to those used by Tracy et al. [13] and Olson et al. [14], with the goal of evaluating associations not only with the primary outcomes of interest (angina and MI), but also with a range of secondary outcomes. As shown by Tracy et al. [13], the immune cell subsets often have skewed distributions. Although Cox models do not require covariates to be normally distributed, the chance that outliers are influential increases when covariate distributions are skewed. If all exposure values have a consistent association with the outcome of interest, then the outlying values do not bias the association of interest. However, if some of the exposure values are outliers due to a separate biological process, then within these outliers there can be an induced association with the outcome of interest that is not causal, and thus biases estimation of the true association of interest. We assume that such a structure exists in the population, rather than being induced by the sampling process. Currently, no method is available to both incorporate the sampling weights and provide robustness in the presence of extreme outliers.

In this paper we extend Bednarski’s partial likelihood method that provides robustness to influential outliers so that it can also incorporate sampling weights. In the “Methods” section we describe our modification to this robust Cox regression method. In the “Simulations” section we illustrate via simulations that this robust method has less bias than traditional weighted Cox regression when a subset of the participants have exposure values that are different from the rest, for reasons that are unrelated to the event of interest, i.e. when there are influential outliers with a different underlying association with outcome. In the “Application” section we evaluate the association between the immune cell traits and stroke in the MESA case-cohort sample to illustrate practical differences between traditional and robust weighted Cox regressions. In the “Discussion” section we compare our weighted robust Cox regression method to alternative estimators.

## Methods

Methods for fitting Cox models that incorporate weighting by inverse sampling probabilities are well-established [2]. However the use of such weighting in combination with robust modeling methods is not consistently implemented. For example, sampling weights are implemented in one robust method that focuses on robustness to variation in proportional hazards (PH) over time [5, 7], but not in another that is more robust to influential outliers [3, 8, 9]. These two methods incorporate a similar approach to robustness, i.e. constructing an estimator with minimum variance subject to a bound on the bias in local neighborhoods of the Cox model, but each considers a slightly different family of estimators. Both lead to partial likelihood estimators that achieve robustness via weighting, so that when sampling weights also exist, the implementations must incorporate both types of weights.

Here we will focus on extending Bednarski’s method, implemented in the R package coxrobust, to generate a new R package coxrobustw that incorporates sampling weights. In simulations and data analysis, we include comparisons to Sasieni and Schemper’s methods, implemented in the R package coxphw. We will refer to Bednarski’s methods as ‘outlier-robust’ and to Sasieni and Schemper’s methods as ‘PH-robust’ due to their focus on robustness to different types of influence on Cox model inferences.

We assume that we have observed data on *n* people, indexed by *i*, each of which has the following values: observed event time *t*_*i*_=min(*T*_*i*_,*C*_*i*_) where $T_{i} \in \mathbb {R}^{+}$ is a potential event time and $C_{i} \in \mathbb {R}^{+}$ is a potential censoring time so that *t*_*i*_∈[0,*T*_*i*_),*z*_*i*_=observed covariate vector, with $z_{i} \in \mathbb {R}^{j}$ where *j* is the number of covariates, and *Δ*_*i*_=$\mathbbm {1}_{\left [{T_{i}<C_{i}}\right ]}$, i.e. 0 for censored observations and 1 for observed events. Core quantities in implementation of partial likelihood estimation for Cox models include *S*^(0)^(*β*,*t*),*S*^(1)^(*β*,*t*),*S*^(2)^(*β*,*t*), and $\bar {z}(\beta,t)$ [15]. Table 1 specifies these quantities for traditional Cox models, Cox models with influence weights as in coxrobust, and Cox models with both coxrobust’s influence weights and sampling weights.

**Table 1:** Key quantities in estimation of Cox model parameters and their variance

Cox PL	Influence Weights	Plus Sampling Weights
$S^{(0)}(\beta,t) = \sum \limits _{j:t_{j} \geq t} e^{\beta ' z_{j}}$	$S_{r}^{(0)}(\beta,t) = \sum \limits _{j:t_{j} \geq t} A(t,z_{j}) e^{\beta ' z_{j}}$	$S_{wr}^{(0)}(\beta,t) = \sum \limits _{j:t_{j} \geq t} w_{j} A(t,z_{j}) e^{\beta ' z_{j}}$
$S^{(1)}(\beta,t) = \sum \limits _{j:t_{j} \geq t} z_{j} e^{\beta ' z_{j}}$	$S_{r}^{(1)}(\beta,t) = \sum \limits _{j:t_{j} \geq t} A(t,z_{j}) z_{j} e^{\beta ' z_{j}}$	$S_{wr}^{(1)}(\beta,t) = \sum \limits _{j:t_{j} \geq t} w_{j} A(t,z_{j}) z_{j} e^{\beta ' z_{j}}$
$S^{(2)}(\beta,t) = \sum \limits _{j:t_{j} \geq t} z_{j} z_{j}' e^{\beta ' z_{j}}$	$S_{r}^{(2)}(\beta,t) = \sum \limits _{j:t_{j} \geq t} A(t,z_{j}) z_{j} z_{j}' e^{\beta ' z_{j}}$	$S_{wr}^{(2)}(\beta,t) = \sum \limits _{j:t_{j} \geq t} w_{j} A(t,z_{j}) z_{j} z_{j}' e^{\beta ' z_{j}}$
$\bar {z}(\beta,t) = \frac {S^{(1)}(\beta,t)}{S^{(0)}(\beta,t)}$	$\bar {z}_{r}(\beta,t) = \frac {S_{r}^{(1)}(\beta,t)}{S_{r}^{(0)}(\beta,t)}$	$\bar {z}_{wr}(\beta,t) = \frac {S_{wr}^{(1)}(\beta,t)}{S_{wr}^{(0)}(\beta,t)}$

Cox partial likelihood estimation uses the score estimating equation $\sum \limits _{i=1}^{n} \left [ z_{i} - \bar {z}(\beta,t_{i}) \right ] = 0$ [1, 16]. The outlier-robust estimator in the coxrobust package uses the modified estimating equation $\sum \limits _{i=1}^{n} A(t_{i}, z_{i}) \left [ z_{i} - \bar {z}_{r}(\beta,t_{i}) \right ] = 0$, where *A*(*t*,*z*) is a smooth non-negative map which is zero for large values of *t* and/or *β*^′^*z*. Note that $\bar {z}$ now has the subscript *r* to indicate that it is a robust version of the weighted mean of the covariate vector, incorporating the function *A*, as defined in Table 1. *A* takes as an input the covariate vector *z* which could contain one or more covariates with influential outliers. As noted by Minder and Bednarski [8], the "double-trimming" accomplished by using A in both parts of the equation leads to the Fisher-consistency of the estimator, so it targets the Cox model parameters. This specific map A is desirable because $\hat {\beta }$ is then Fréchet differentiable yielding a consistent and asymptotically normal estimator of *β* for infinitesimal extensions of the Cox model [3] [lemmas 4.2 & 4.3], the definition of outlier-robustness used by Bednarski.

The outlier-robust estimator described in the previous paragraph relies on mathematical details in Bednarski’s 1993 paper [3]. Highlights from that paper are included here for clarity. Bednarski’s outlier-robust estimator relies on writing the Cox score estimating equation in terms of the empirical distribution function *F*_*n*_(*t*,*c*,*z*) of the sample (*T*_1_,*C*_1_,*Z*_1_),...,(*T*_*n*_,*C*_*n*_,*Z*_*n*_):

$${\begin{aligned} {\int \left[y - \frac{\int z \mathbbm{1}_{\left[ {(a \wedge t) \geq w} \right] } \exp(\beta'z)dF_{n}(t,a,z)}{\int \mathbbm{1}_{\left[ {(a \wedge t) \geq w} \right] } \exp(\beta'z)dF_{n}(t,a,z)}\right] \mathbbm{1}_{\left[ {w \leq c} \right] }dF_{n}(w,c,y) = 0.} \end{aligned}} $$

This equation is modified with a class $\mathcal {A}$ of smooth functions from $\mathbb {R}^{+} \times \mathbb {R}^{j} \rightarrow \mathbb {R}^{+}$ to give a modified class of regression parameter estimators, defined by the vector equation *L*(*F*_*n*_,*β*,*A*)=

$${\begin{aligned} \int A(w,y) \left[y - \frac{\int A(w,z) z \mathbbm{1}_{\left[(a \wedge t) \geq w\right]} \exp(\beta'z)dF_{n}(t,a,z)}{\int A(w,z) \mathbbm{1}_{\left[(a \wedge t) \geq w\right]}\exp(\beta'z)dF_{n}(t,a,z)} \right] \mathbbm{1}_{\left[w \leq c\right]} dF_{n}(w,c,y) = 0 \end{aligned}} $$

for *A* in $\mathcal {A}$. Bednarski uses functions *A* yielding Fréchet differentiable functionals *L*(*F*_*n*_,*β*,*A*) that give Fisher-consistent estimators of Cox model parameters (Lemma 3.1 in [3]).

Bednarski [3] goes on to specify the conditions that are necessary for $\sqrt {n}$-consistency, Fréchet differentiability, and asymptotic normality (Theorems 4.1-4.3) in the case without censoring, which can be extended to incorporate censoring. For *B* a closed set in $\mathbb {R}^{j}$ containing an open neighborhood of the true parameter *β*_0_, *F* the true distribution of t and z from the Cox model distribution, $\mathcal {A}^{*}$ a class of functions from $\mathbb {R}^{+} \times \mathbb {R}^{j} \rightarrow \mathbb {R}^{+}$, *A*_0_ a non-negative continuous function of time with bounded support *S*_*b*_=[*a*,*b*], and $\mathcal {A}$ a class of functions $\{A_{0} A; A \in \mathcal {A}^{*}\}$, the following conditions need to hold:

- (A1) For all $A \in \mathcal {A}^{*}$ and $w \in S_{b}, \int A(w,z) \mathbbm {1}_{\left [ {t \geq w} \right ] } dF(t,z) > \epsilon $ for some *ε*>0.
- (A2) All the functions from $\mathcal {A}^{*}$ vanish outside a bounded set, they are absolutely continuous and have jointly bounded variation. The set $\mathcal {A}^{*}$ is compact for the supremum norm on $C(\mathbb {R}^{j}\times \mathbb {R}^{+})$, i.e. the space of continuous functions from $\mathbb {R}^{j} \times \mathbb {R}^{+}$ to $\mathbb {R}^{+}$.
- (A3) The following functions of variables $(w,y) \in \mathbb {R}^{+} \times \mathbb {R}^{j}$:
  $$\begin{array}{*{20}l} {A(w,y) \frac{\int A(w,z) z \mathbbm{1}_{\left[ {t \geq w} \right] } \exp(\beta_{0}'z) dF(t,z)}{\int A(w,z) \mathbbm{1}_{\left[ {t \geq w} \right] } \exp(\beta_{0}'z) dF(t,z)}} \end{array} $$
  have jointly bounded variation for $A \in \mathcal {A}$ and *β*∈*B*.

In the case when censoring is present, indicators $\mathbbm {1}_{\left [ {t \geq w} \right ] }$ become $\mathbbm {1}_{\left [ {(a \wedge t) \geq w} \right ] }$ and the inner integration is with respect to *F*(*t*,*a*,*z*). The function *A*(*w*,*y*) is multiplied in the outer integral by $\mathbbm {1}_{\left [w \leq c\right ]}$ and the integral is with respect to *F*(*w*,*c*,*y*).

Specifically, in the coxrobust implementation, the map *A* is *A*_*β*,*M*_(*t*,*z*)=*M*−min(*M*,*t* exp(*β*^′^*z*)), where *M* is an order statistic in the sample *t*_1_ exp(*β*^′^*z*_1_),...,*t*_*n*_ exp(*β*^′^*z*_*n*_). The class of functions $\mathcal A^{*}$ is the set {*A*_*β*,*M*_:*β*∈*B*,*M*≤*M*^∗^}, where *M*^∗^ is some fixed upper bound for *M*. In the default implementation, *M* is the 95^*th*^ percentile, but the percentile is a modifiable input to the R function. *A* and *β* are estimated iteratively, with three iterations leading to convergence in the scenarios they examined, and thus three iterations implemented in the coxrobust package [3]. To incorporate sampling weights, we added a step after the estimation of *A* and *β* that incorporates the sampling weights *w* [11] into the estimating equation $\sum \limits _{i=1}^{n} w_{i} A(t_{i}, z_{i}) \left [ z_{i} - \bar {z}_{wr}(\beta,t_{i}) \right ] = 0$, with the details of the modified weighted mean covariate vector $\bar {z}_{wr}$ given in Table 1 [15]. The reason for adding sampling weights after iteration is so that the influence weights reflect influence due to outliers, rather than due to large sampling weights.

In addition to deriving a consistent estimator of *β*, Bednarski [3] also derives an influence function that can be used to approximate the estimator’s variance, both at the model and at small departures from it. The existence of this influence function relies on sufficient smoothness of *A*, as discussed in section 5 of his 1993 paper. The resulting variance estimate for the specific choice of *A* implemented in coxrobust is:

1 $$\begin{array}{*{20}l} \hat{V}_{r}(\hat{\beta}) &= I_{r}^{-1}(\hat{\beta}) \left[ \sum\limits_{i=1}^{n} [r_{i}(\hat{\beta})] [ r_{i}(\hat{\beta})]' \right] I_{r}^{-1}(\hat{\beta})  \end{array} $$

where $I_{r}(\hat {\beta })$ is the observed information matrix that incorporates outlier downweighting:

$${\begin{aligned} I_{r}(\hat{\beta}) &= \sum\limits_{i=1}^{n} \Delta_{i} A(t_{i},z_{i}) \frac{S_{r}^{(0)}(\hat{\beta},t_{i}) S_{r}^{(2)}(\hat{\beta},t_{i}) - \left[ S_{r}^{(1)}(\hat{\beta},t_{i}) \right] \left[ S_{r}^{(1)}(\hat{\beta},t_{i}) \right]'}{\left[ S_{r}^{(0)}(\hat{\beta},t_{i}) \right]^{2}} \end{aligned}} $$

and $r_{i}(\hat {\beta })$ is a residual for the *i*th subject:

$${\begin{aligned} r_{i}(\hat{\beta}) &= \Delta_{i} A(t_{i},z_{i}) \left[ z_{i} - \bar{z}_{r}(\hat{\beta},t_{i}) \right] \\ & \hspace{0.5in} - \sum\limits_{k:t_{k} \geq t_{i}} \frac{\Delta_{k} A(t_{i},z_{k}) A(t_{k},z_{k}) \exp(\hat{\beta}'z_{k})}{S_{r}^{(0)}(\hat{\beta},t_{k})} \left[ \bar{z}_{r}(\hat{\beta},t_{k}) - z_{k} \right] \text{.} \end{aligned}} $$

This specific formulation of the variance estimate does not apply to all possible specifications of *A*, but does apply to the one chosen by Bednarski for the coxrobust package and implemented in this paper.

The variance estimate for the new coxrobustw algorithm incorporates the sampling weights *w* into the jackknife variance estimate in Eq. (1), i.e.

$$\begin{array}{*{20}l} \hat{V}_{wr}(\hat{\beta}) &= I_{wr}^{-1}(\hat{\beta}) \left[ \sum\limits_{i=1}^{n} [r_{i}^{wr}(\hat{\beta})] [ r_{i}^{wr}(\hat{\beta})]' \right] I_{wr}^{-1}(\hat{\beta})  \end{array} $$

where $I_{wr}(\hat {\beta })$ is the observed information matrix that incorporates both sampling weights and outlier downweighting:

$${\begin{aligned} I_{wr}(\hat{\beta}) &= \sum\limits_{i=1}^{n} \Delta_{i} w_{i} A(t_{i},z_{i}) \frac{S_{wr}^{(0)}(\hat{\beta},t_{i}) S_{wr}^{(2)}(\hat{\beta},t_{i}) - \left[ S_{wr}^{(1)}(\hat{\beta},t_{i}) \right] \left[ S_{wr}^{(1)}(\hat{\beta},t_{i}) \right]'}{\left[ S_{wr}^{(0)}(\hat{\beta},t_{i}) \right]^{2}} \end{aligned}} $$

and $r_{i}^{wr}(\hat {\beta })$ is a residual for the *i*th subject:

$${\begin{aligned} r_{i}^{wr}(\hat{\beta}) &= \Delta_{i} w_{i} A(t_{i},z_{i}) \left[ z_{i} - \bar{z}_{wr}(\hat{\beta},t_{i}) \right] \\ & \hspace{0.4in} - \sum\limits_{k:t_{k} \geq t_{i}} \frac{\Delta_{k} w_{i} A(t_{i},z_{k}) w_{k} A(t_{k},z_{k}) \exp(\hat{\beta}'z_{k})}{S_{wr}^{(0)}(\hat{\beta},t_{k})} \left[ \bar{z}_{wr}(\hat{\beta},t_{k}) - z_{k} \right] \text{.} \end{aligned}} $$

The new R package coxrobustw implements this robust estimator that incorporates sampling weights, using the modified score equation and variance estimate detailed above. As in the original package, the algorithm uses three iterations and a default M of the 95^*th*^ percentile. The package is publicly available at https://github.com/csitlani/coxrobustw.

## Results

### Simulations

We conducted simulations to illustrate the utility of this new weighted robust Cox regression procedure. For both a complete population, and a case-cohort sample from that population, we compared traditional Cox regression model estimates to robust Cox regression model estimates using our new package coxrobustw (‘outlier-robust’) and the existing coxphw that is robust to departures from proportional hazards (‘PH-robust’). For the case-cohort sample, the weighted versions of all three methods were used, with weights being the inverse of the sampling probability. Two versions of the outlier-robust method were included, with the truncation parameter M set to either the 90^*th*^ or the 95^*th*^ percentile.

We generated time to event data by specifying the hazard ratio associated with a one-unit difference in exposure *x* (HRx), which was incorporated into the scale parameter of a Weibull distribution, i.e. scale = 1000× exp(− ln(HRx)×*x*) and shape = 1. The censoring time also had a Weibull distribution, with scale = 2 and shape = 1, and the observed time was set to be the minimum of the censoring time and the time to event. We generated exposure data from a normal distribution, with mean and variance described below, but truncated the values at 0 and 100 to mirror the type of exposure data available in the MESA immune cell trait project. Immune cell traits were analyzed as a percentage of their parent population, e.g. Th1 cells were analyzed as a percentage of CD4+ cells. Contamination was subsequently added by changing the mean of the exposure distribution for a fixed portion of the observations. For example, we simulated an exposure with mean 12 and standard deviation (SD) 8, from which the survival data were generated based on an assumed HRx of 1.25. Then for a portion of the observations, we replaced the exposure data with data from a normal distribution with higher mean, but still SD 8. The scenarios in Table 2 include no contamination, 5% contamination with mean 24, 5% contamination with mean 36, and 10% contamination with mean 24. We evaluated the methods both on the full sample of n=6000 people, and on the case-cohort sample that was generated by keeping all people who experienced an event, plus a random sample of size 600 from those who did not experience an event. The average size of the case-cohort sample was 1080, and the average percent of contaminated observations in the sample matched the specified level of contamination, regardless of whether or not a weighted percentage was calculated. All simulations were conducted in R version 3.2.3 [17], and were repeated one thousand times for each setup.

**Table 2:** Mean coefficient estimates (and mean standard errors) from 1000 simulations

		Normal	5% 2x mean	5% 3x mean	10% 2x mean
Population	Cox PL	0.223 (0.007)	0.141 (0.005)	0.081 (0.003)	0.118 (0.005)
(unweighted)	PH-robust	0.224 (0.021)	0.130 (0.015)	0.074 (0.009)	0.106 (0.013)
	outlier-robust90	0.224 (0.015)	0.204 (0.012)	0.175 (0.009)	0.185 (0.011)
	outlier-robust95	0.224 (0.014)	0.200 (0.011)	0.163 (0.008)	0.179 (0.009)
Sample	Cox PL	0.225 (0.011)	0.149 (0.015)	0.084 (0.009)	0.123 (0.012)
(weighted)	PH-robust	0.233 (0.024)	0.150 (0.022)	0.084 (0.014)	0.121 (0.020)
	outlier-robust90	0.224 (0.021)	0.192 (0.015)	0.152 (0.010)	0.171 (0.013)
	outlier-robust95	0.224 (0.025)	0.189 (0.017)	0.144 (0.010)	0.167 (0.014)

The true value of the coefficient *β* is log(1.25)=0.223. Two versions of the outlier-robust method are included: one using truncation parameter M=0.90 (outlier-robust90) and the other using M=0.95 (outlier-robust95)

The results in Table 2 show that both the traditional Cox model and the robust versions provide essentially unbiased estimates when no contamination is present, i.e. the mean coefficient estimate is approximately log(1.25)=0.223. As expected, the traditional model is more efficient than any of the robust methods when modeling assumptions are satisfied. However, when contamination is present, all methods are biased toward the null. The traditional Cox model and the PH-robust method are substantially more biased than the outlier-robust method because they do not incorporate methods to detect and minimize the influence of outliers. The outlier-robust version, on the other hand, uses the map *A* specified in the “Methods” section and implemented in the coxrobust package, along with its truncation parameter *M*, to minimize the influence of the contaminated observations.

The true parameter value is not recovered by the outlier-robust method in part because the accuracy of outlier detection is not consistently high. Outlier detection metrics are not straightforward, due to the use of *A* both at the observation level and in contributions to the weighted covariate mean $\bar {z}_{wr}$ at each event time. However, averaging over event times and simulations, for the three contamination scenarios considered in this paper, the percentage of contaminated observations correctly identified as such varies from 30 to 95%. Likewise, the percentage of correctly discarded observations varies from 24 to 83%. Outlier detection was similar in the population and in the sample. Comparing choice of truncation parameter, higher sensitivity for detecting contaminated observations corresponds to lower percentage of correctly discarded observations. On balance, using the 90^*th*^ percentile as the truncation parameter in the outlier-robust method yields similar, but slightly less biased, estimates of association, when compared to using the 95^*th*^ percentile. The (unweighted) population results are qualitatively similar to the weighted results for the case-cohort sample. The weighted results use the new coxrobustw package.

### Application

To illustrate the use of robust weighted Cox methods in data obtained from human subjects, we analyzed a secondary outcome in the MESA case-cohort study of immune cell traits. Specifically, we examined occurrence of stroke as the outcome event and 17 immune cell traits postulated to be associated with cardiovascular disease as the exposures of interest. The immune cell traits were quantified as percent of total immune cells, or percent of a subset of immune cells. Table 3 describes the specific measures that were used. Based on a review of the literature, often in animal models, the lymphocyte subsets cluster into four groups: 1) high levels of pro-inflammatory cells; 2) high levels of pro-fibrotic cells; 3) high levels of anti-inflammatory and anti-fibrotic cells; and 4) high levels of pro-inflammatory cells that mark chronic use of adaptive immunity. All clusters are thought to increase cardiovascular risk except for the third, which is thought to decrease it [18–21]. The primary goal of this analysis was to illustrate the use of the new statistical method, rather than to draw key conclusions about the associations between the immune cell traits and stroke events.

**Table 3:** Definitions of the immune cell traits

	Cell surface and intracellular markers
**Pro-inflammatory cells**	
T helper type 1 (Th1) cells	CD4+IFN+ (expressed as a % of CD4+ cells)
T helper type 17 (Th17) cells	CD4+IL17+ (expressed as a % of CD4+ cells)
Activated CD4+ cells	CD4+CD38+ (expressed as a % of CD4+ cells)
Activated CD8+ cells	CD8+CD38+ (expressed as a % of CD8+ cells)
Natural Killer (NK) cells	CD3-CD56+CD16+ (expressed as a % of lymphocytes)
Gamma delta T cells	CD3+ *γδ*TCR+ (expressed as a % of CD3+ cells)
Classic Monocytes	CD14++CD16- (expressed as a % of monocytes)
**Pro-fibrotic cells**	
T helper type 2 (Th2) cells	CD4+IL4+ (expressed as a % of CD4+ cells)
Non-classic Monocytes	CD14+CD16++ (expressed as a % of monocytes)
**Anti-inflammatory and anti-fibrotic cells**	
T regulatory cells (T-reg)	CD4+CD25+CD127- (expressed as a % of CD4+ cells)
Intermediate Monocytes	CD14+CD16+ (expressed as a % of monocytes)
**Pro-inflammatory cells that mark chronic use of adaptive immunity**	
Naive CD4+ cells	CD4+CD45RA+ (expressed as a % of CD4+ cells)
Naive CD8+ cells	CD8+CD45RA+ (expressed as a % of CD8+ cells)
Senescent CD4+ cells	CD4+CD28- (expressed as a % of CD4+ cells)
Senescent CD8+ cells	CD8+CD28- (expressed as a % of CD8+ cells)
CD4+ memory cells	CD4+CD45RO+ (expressed as a % of CD4+ cells)
CD8+ memory cells	CD8+CD45RO+ (expressed as a % of CD8+ cells)

The entire MESA cohort is a racially diverse cohort of 6814 adults between the ages of 45 and 84 years enrolled between 2000 and 2002 from six field centers across the United States. The MESA protocol has been approved by the Institutional Review Boards of all collaborating institutions, and all participants gave informed consent. Cryopreserved blood samples from the baseline visit were assayed at the University of Vermont to measure lymphocyte and monocyte subsets, using methods similar to those used by Tracy et al. [13] and Olson et al. [14]. From participants who had two vials of cryopreserved cells, a random cohort of 765 participants was sampled, along with all additional cases of MI and angina, for a total sample size of 1200 participants. Participants were followed for stroke outcomes through 2015. In order to ensure that estimates can be generalized to the MESA population, the sampling design necessitates use of sampling weights in statistical models, even for secondary outcomes such as stroke. The weighted mean age of participants included in this analysis was 62 years, and 54% were male. The sample was 39% White, 28% Black, 21% Hispanic, and 12% Chinese American. Stroke events occurred in 6% of the sample (N=70), which corresponds to a weighted rate of 4.6% in the population.

A number of the immune cell traits have outliers, implying the potential usefulness of robust methods that minimize their influence on association estimates. Summary data provided in Table 4 illustrate that the maximum value is often several SDs or more above the mean.

**Table 4:** Summary data for the 17 immune cell traits in the MESA case-cohort study

	N	Mean	SD	Min	Max
**Pro-inflammatory cells**					
T helper type 1 (Th1) cells	770	15.3	9.0	0.0	65.5
T helper type 17 (Th17) cells	770	2.1	1.4	0.0	15.7
Activated CD4+ cells	1051	26.1	12.1	4.1	77.0
Activated CD8+ cells	1062	23.6	12.2	2.2	71.8
Natural Killer (NK) cells	1087	5.0	5.7	0.0	33.7
gamma delta T cells	1087	6.6	6.1	0.3	57.7
Classic Monocytes	922	74.4	10.2	9.3	96.3
**Pro-fibrotic cells**					
T helper type 2 (Th2) cells	770	2.9	1.7	0.0	11.9
Non-classic Monocytes	922	7.4	7.5	0.0	81.4
**Anti-inflammatory and anti-fibrotic cells**					
T regulatory cells (T-reg)	1035	5.0	2.2	0.0	15.2
Intermediate Monocytes	922	18.1	7.1	3.0	46.0
**Pro-inflammatory cells that mark chronic use of adaptive immunity**					
Naive CD4+ cells	1051	26.1	12.0	1.6	70.8
Naive CD8+ cells	1062	52.4	14.7	6.5	97.1
Senescent CD4+ cells	1051	13.9	10.0	1.0	69.1
Senescent CD8+ cells	1062	55.6	15.9	10.4	94.0
CD4+ memory cells	1051	51.7	13.4	12.8	86.7
CD8+ memory cells	1062	21.7	10.6	0.0	79.2

Analyses were performed using several methods: traditional Cox models, traditional Cox models after Winsorizing the exposure at 4 SDs from the mean [22], and both weighted robust methods (outlier-robust coxrobustw and PH-robust coxphw). The truncation parameter M was set at the 95^*th*^ percentile, with sensitivity analyses performed using the 90^*th*^ percentile. Confidence intervals based on sandwich variance estimates were used throughout to account for the inverse-probability of sampling weights. Separate models were fitted for each of the immune cell traits, without adjustment for other traits. A conservative approach of Bonferroni correction [23] was used to account for the 17 immune cell traits. Estimated hazard ratios are per SD of the percent of each immune cell type.

Due to the small number of stroke events, we included limited adjustment for covariates. Specifically, baseline age, gender, and race/ethnicity (White, Black, Hispanic, Chinese American) were included as adjustment variables in the regression models. Based on previous analyses of stroke in the MESA data [24], we ran sensitivity analyses that included additional covariates such as season of blood draw, systolic blood pressure, cardiovascular medications (anti-hypertensives and statins), smoking, education (via an indicator of having attained a bachelor’s degree or higher), low-density lipoprotein cholesterol, total cholesterol, diabetes, and body mass index.

**Fig. 1:**
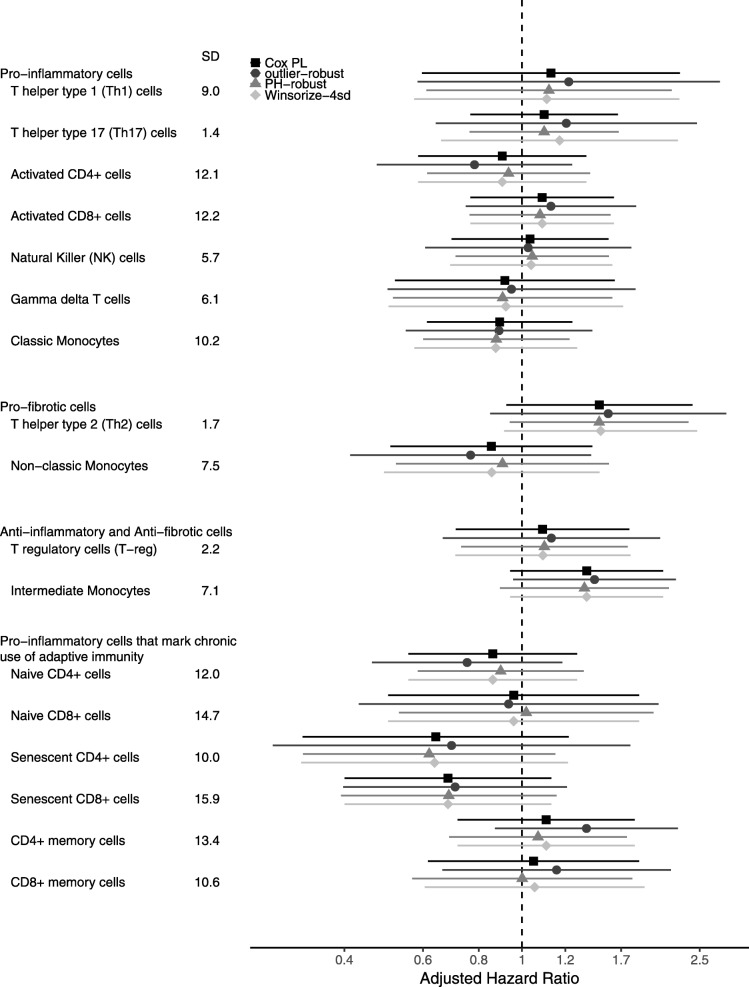
Stroke and immune cell trait associations in MESA. Estimated hazard ratios, per SD of immune cell subset, and 99.7% confidence intervals (to incorporate Bonferroni correction for 17 tests) for associations between risk of stroke and immune cell subsets

After correction for multiple testing, there were no significant associations between stroke and the immune cell traits (Figure 1). Given that there were only 70 stroke cases, the power to find an association was small, so the lack of clinically important conclusions is not surprising, but our focus is on the comparative results across methods. Consistent with our simulations, traditional Cox methods and the PH-robust method gave estimates that were more similar to each other than to the outlier-robust method. Traditional methods using Winsorization were quite similar to those without Winsorization, and were thus different from the outlier-robust method. This difference was not surprising, given the different levels of truncation in each method (95^*th*^ percentile versus ±4 SDs) and the incorporation of both exposure value and time-to-event in the outlier-robust method versus just exposure value in Winsorization. Notably, when the outlier-robust method differed from traditional Cox methods, it most often gave point estimates further from the null, consistent with the idea that the outliers may be the result of an unrelated process that leads to attenuated association estimates obtained with non-robust methods. The outlier-robust method generated wider confidence intervals than the traditional Cox method, which is to be expected given the added robustness to influential outliers. Results were similar when additional adjustment covariates were added to the models or the truncation parameter M was set to the 90^*th*^ percentile.

## Discussion

This paper extends Cox proportional hazards regression methods that are robust to outliers in exposure data, so that they also incorporate sampling weights. When outliers are not causally related to the outcome of interest in the same way that other exposure values are, the outlier-robust method provides a less biased estimate of the true association than traditional methods. One application for this weighted outlier-robust method is in a case-cohort sample where the exposure of interest contains outliers; we provided such an example in a MESA case-cohort study where immune cell traits were measured. No significant associations with stroke events were found using traditional Cox models. Both in the scenario we simulated and in the illustrative dataset, larger associations were most often seen using the outlier-robust method, which supports the idea that traditional methods may underestimate associations. That said, the relative results will depend on the specific contamination model, and cannot be generalized to all possible scenarios based on the illustrations we provide.

Although normality is not required for covariates in Cox models, some departures from normality, such as skewness, lead to an increased chance of influential outliers. In cases of contamination such as those described in this paper, the skewness can reflect a source of bias in estimation of the exposure-outcome association. One method to minimize this bias is the outlier-robust one we have described. Alternative methods for analyzing exposure data that are not normally distributed include artificially truncating or Winsorizing [22], as well as transforming the data. We have shown in our application that Winsorizing the exposure data at 4 SDs from the mean generally does not substantially change the estimates. In simulation data not shown, Winsorizing at 2, 3, or 4 SDs from the mean still resulted in a more biased association estimate than using the weighted robust method, and the corresponding variance estimate did not account for the modification to the data. Log-transformation would make the exposure distribution less skewed, while maintaining reasonable interpretation; however, it would not incorporate the idea that the outliers are there for an external reason, and thus are not related to the outcome in the same way that other observations are. Consideration of alternatives emphasizes the idea that the source of the outliers is important, and the choice of method may depend on the reason outliers exist.

Specifically, different approaches might be warranted if the outliers are the result of a separate biological process, rather than being technical artifacts. For example, if a participant has a damaged blood sample or there is a technical malfunction of the flow cytometer used to obtain immune cell traits, then omitting the incorrect data is likely warranted. Truncation may be a better option for less well-defined technical artifacts that are recognized not to be plausible true values. On the other hand, in the case where the outliers are the real product of a biological process, for example if a participant has an undetected human immunodeficiency virus infection which has led to a low (or even zero) T helper type 1 (Th1) cell count, then outlier-robust methods, such as the one proposed here, are most appropriate.

The type of sampling would also affect the most appropriate use of weighting in a robust Cox regression approach. This paper focused on outcome-based sampling, given covariates that have outlying values that are in some sense wrong (atypical for the individual, assay errors, etc). These covariates were not used to choose the subsample; in fact, they were only measured on the subsample. We would expect that the true values of the covariates for these individuals would be related to the sampling weights, because the true values would be related to risk. However, conditional on risk, the outlying values would not be related to the sampling weights. Because sampling is not based on the outlying covariates, there is no harm in detecting outliers based on the sample, rather than reweighting to the full cohort. There is potentially harm in detecting outliers after reweighting, because the outlier threshold will be excessively sensitive to values in the reference subcohort. Under other sampling schemes it might well be preferable to modify the current procedure to include sampling weights in the influence iteration.

## Conclusions

Adding sampling weights to robust Cox regression methods provides a new tool to analyze time-to-event data with sampling, e.g. case-cohort data, when exposures of interest contain outliers. A readily available R package facilitates implementation of this new method.

## Data Availability

The data that support the findings of this study cannot be publicly posted due to identifiability concerns. They are however available upon reasonable request from the data coordinating center for the MESA Cohort study (https://www.uwchscc.org/) or, eventually, via BioLINCC (https://biolincc.nhlbi.nih.gov/home/).

## Abbreviations

BioLINCC: Biologic Specimen and Data Repository Information Coordinating Center
DSMB: Data and Safety Monitoring Board
FWA: Federalwide Assurance
IRB: Institutional Review Board
MESA: Multi-Ethnic Study of Atherosclerosis
MI: myocardial infarction
NCATS: National Center for Advancing Translational Sciences
NHLBI: National Heart, Lung, and Blood Institute
NK: Natural Killer
PH: proportional hazards
T-reg: T regulatory cells
Th1: T helper type 1
Th17: T helper type 17
Th2: T helper type 2
